# Functional High-Intensity Interval Training Lowers Body Mass and Improves Coordination, Strength, Muscular Endurance, and Aerobic Endurance of Inmates in a German Prison

**DOI:** 10.3389/fphys.2021.733774

**Published:** 2021-09-30

**Authors:** Milan Dransmann, Martin Koddebusch, Bernd Gröben, Pamela Wicker

**Affiliations:** Department of Sports Science, Bielefeld University, Bielefeld, Germany

**Keywords:** prison, male, body composition, fitness, circuit training, high-intensity interval training, high-intensity functional training

## Abstract

This study examined the effects of circuit-like functional high-intensity interval training (HIIT) on body composition and motor performance of inmates in an open German prison. The group of inmates (*n*=11) consisted of predominantly overweight males [average body-mass-index (BMI)=31.2]. They performed 6weeks of training including 3 sessions per week. The 6-week training program was framed by a pre-test and a post-test that assessed anthropometry and motor performance. On average, the inmates participated in 91.9% of all training sessions. The intervention significantly lowered body mass (*p*=0.007) and BMI (*p*=0.006). Fat mass and fat-free mass did not change significantly from pre-test to post-test. The times in 20m sprint did not change. The performance in lateral jumping from side-to-side (*p*=0.024), standing long jump (*p*=0.001), and 30–15 Intermittent Fitness Test (*p*<0.001) improved significantly. The greatest improvements were observed in the number of sit-ups (*p*<0.001) and push-ups (*p*<0.001). These findings suggest that (functional) HIIT is a practical and effective training method in the context of a prison.

## Introduction

Prison populations are growing in many Western countries (Pérez-Moreno et al., 2007). The overriding goal of prisons is resocialization and sport is considered to play a significant role in resocialization. Sport activities have a high priority in prisons and represent the most popular leisure activity of inmates (Kuhn, 2019). Participation in sports allows inmates to overcome the monotony and boredom of daily prison routines (Müller and Mutz, 2019). Nevertheless, studies examining the impact of sport-based interventions within prison populations were described as limited (Meek and Lewis, 2014) and embryonic (Gallant et al., 2015).

A few studies have examined outcomes of sport-based interventions in prisons. For example, Gettman et al. (1976) studied running programs of different frequencies and reported significant improvements in maximum oxygen uptake, resting and recovery heart rates, maximum treadmill stress test, oxygen pulse, and pulmonary ventilation. The inmates in Pérez-Moreno et al. (2007), who were HIV/HVC co-infected and enrolled in a methadone maintenance program, could enhance their cardiorespiratory fitness in a gradual cycle ergometer test and their dynamic strength. Male inmates with chronic illness in an Australian prison (Cashin et al., 2008) have achieved significant improvements in resting heart rate and aerobic endurance (6-min walk test). Battaglia et al. (2013) showed that a cardiovascular and resistance training (CRT) is more effective than a high-intensity strength training (HIST): performance in a 10 × 5 shuttle run test, dynamic strength endurance, and estimated maximum oxygen uptake improved as a result of both types of training, while the body-mass-index (BMI) decreased only through CRT.

What all four studies have in common is that they were time-consuming, both in terms of the length of a session (minimum 1h) and the overall intervention (minimum 12weeks). It is possible that the large amount of time required explains the low adherence to the training programs: 57% in Battaglia et al. (2013) and 71% in Pérez-Moreno et al. (2007). Also, the mentioned studies (except for Gettman et al., 1976) use a variety of materials in training. The intensive reliance on training equipment is challenging because exercise equipment is usually difficult to access in prisons (Ghanbarzadeh and Mohamadi, 2012). Consequently, given the time and equipment constraints of a prison, an effective training method is required that has an attractive time-use ratio and does not require any material by using bodyweight exercises.

High-intensity interval training (HIIT) represents such a possible method in the prison context. This method became popular due to its attractive time-use ratio and is characterized by unimodal movements like running (Dransmann, 2020). By now, relatively novel variations of the traditional HIIT have been developed that maintain HIIT characteristics in terms of training parameters, but change the focus from purely endurance training to more functional and strength-oriented training protocols (Sperlich et al., 2018). Due to the different responses evoked by endurance and strength, the functional HIIT leads to synergistic improvements in both cardiorespiratory and metabolic parameters (Sperlich et al., 2017). While cardiorespiratory fitness is associated with improved health and less premature death (Bouchard et al., 2015), strength exercise is related to elevated muscle mass and improved body composition (Poehlman et al., 2000). These beneficial health outcomes are particularly relevant for inmates, as imprisoned people usually have a poor health status compared with the general population (Mannocci et al., 2015).

Functional HIIT programs are not as widely explored as the traditional HIIT protocols. The circuit-like functional high-intensity training of Sperlich et al. (2017) increased the peak oxygen uptake, lowered body mass, decreased BMI, reduced fat mass, enhanced fat-free mass, and improved functional strength of overweight women. The overweight women trained 9weeks with three sessions per week of an average duration of around 30min. A mobile based Circuit_HIIT_ performed once or twice per day by untrained individuals just improved the functional strength, whereas body mass, fat mass, fat-free mass, and peak oxygen uptake did not change (Sperlich et al., 2018). The authors explained this finding by the short duration of the sessions (6min each) and the shortness of the intervention (4weeks).

A functional HIIT should not be confused with a high-intensity functional training (HIFT). HIFT workouts are based on completing a certain number of repetitions in the fastest time possible or on completing as many repetitions as possible within a given time frame for a series of exercises. Thus, HIFT protocols are based on the exclusion of defined rest intervals – in contrast to more traditional HIIT protocols (Feito et al., 2018).

A functional HIIT has not yet been investigated in the context of a prison. Accordingly, this study examined the effects of a functional HIIT program on anthropometric and motor performance parameters among a group of inmates. The group of inmates was composed of male, German offenders who were serving their prison sentence in a German penitentiary. Following the presented findings of scientific research, our hypothesis was that the applied functional HIIT program improved body composition (body mass, BMI, fat mass, and fat-free mass) and motor performance (muscular endurance and aerobic endurance) of participants.

## Materials and Methods

### Participants

The study started with 20 male offenders. Participants served their imprisonment in an open prison, which means that they were free to leave the penitentiary for work, school, or formation. Depending on the behavior in prison and the current detention level, individual releases for a few hours or days were also possible. During the study period, prisoners were – due to the Corona pandemic – only allowed to leave the prison for work.

Thirteen of the originally 20 participants completed the intervention, whereas seven participants dropped out. One participant was transferred to another facility, another was released early on parole, and two others escaped. Another two participants could not complete the program due to medical reasons and one was disqualified because of exceeding the maximum absenteeism rate (i.e., four sessions).

After the post-test 2, further participants had to be excluded from the empirical analysis. High increases in body mass (+7.9 and +6.7kg) and BMI (+2.4 and +2.2kg·m^2^) were witnessed among these two inmates. Since these changes were counterintuitive, the researchers scheduled follow-up meetings with problem centered interviews. In these interviews, both inmates revealed their drug consumption (marijuana and amphetamines) and a lack of regulated food intake before imprisonment. Their pre-test was 2 and 4weeks after their individual detention. The values of both participants would affect the distribution of variables and increase the variation in anthropometric parameters, ultimately biasing the findings of the other inmates. Since, in addition, a negative correlation between BMI and motor performance of young adults was found (Jiménez et al., 2016); these two participants were excluded from the whole empirical analysis.

Consequently, 11 participants (age 25.82±5.25) were included in the empirical analysis. Since the average BMI of the test participants was 31.21±7.38, the test group can be classified as obese following the classification of the WHO (WHO, 2021). Nonetheless, three participants were classified as normal weight, three participants were classified as overweight, one participant was classified as having obesity class I, and two participants were classified as having class II and class III obesity, respectively (WHO, 2021).

Before the start of the program, all test persons were informed about the study’s design and content, about data handling, and the right to withdraw from the study before providing their consent in written form. The inclusion criteria were a completion of more than 80% of the training sessions, a health status allowing individuals to engage in physical activity (was determined by the management of the institution), no daily intake of medication, no intake of drugs, and lack of other training during the study period. A physical examination was not conducted and physician clearance was not given either. All procedures were conducted in accordance with the Declaration of Helsinki. The training protocol was approved by the ethical review board of Bielefeld University.

### Overall Study Design

**Figure 1:**
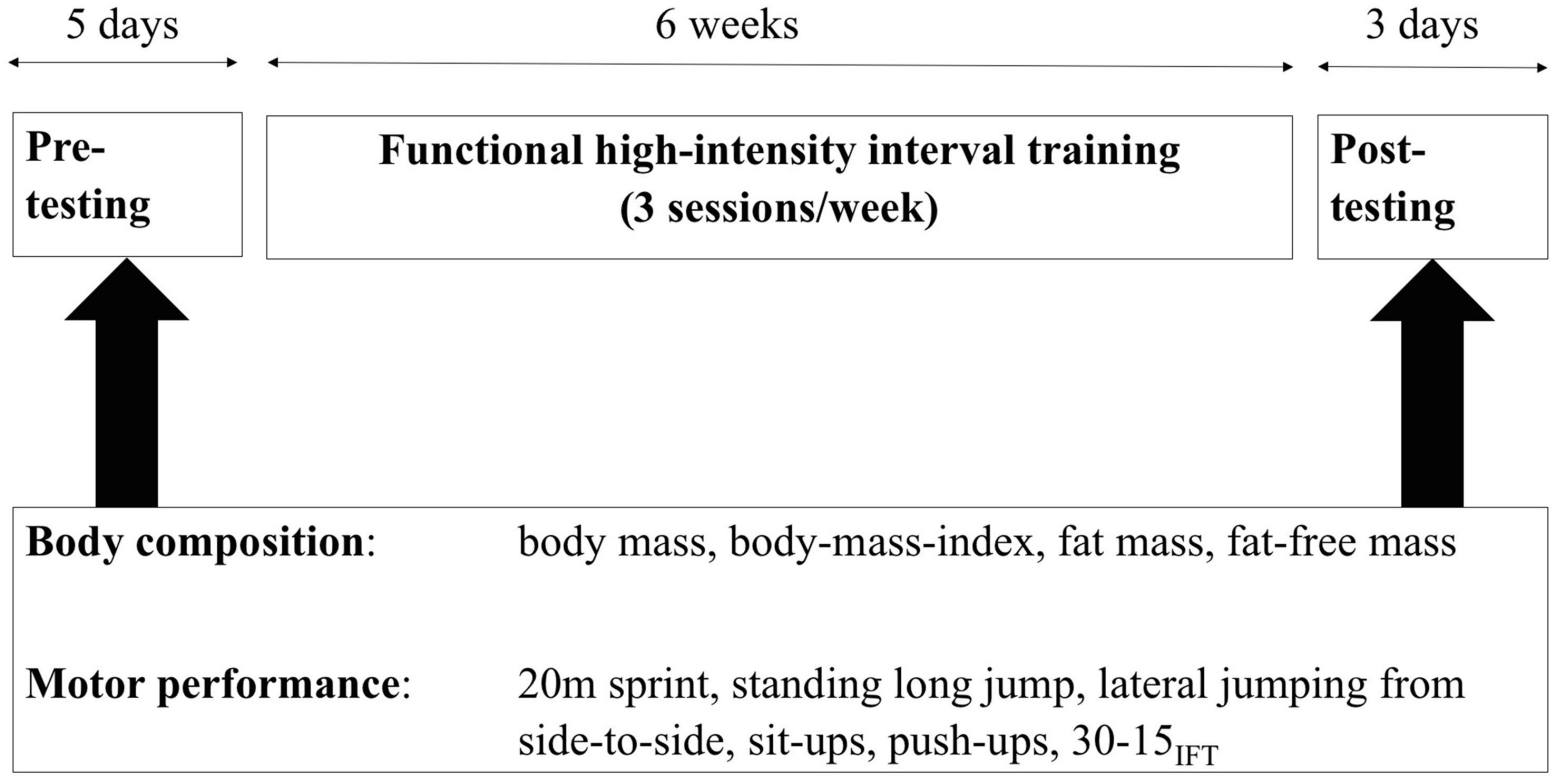
The timeline and outcome variables of this study.

The overall study consisted of a HIIT intervention, which was framed by a pre-test and a post-test. The intervention comprised 6weeks of training with three sessions each week on Mondays, Wednesdays, and Fridays. Figure 1 gives an overview of the overall study design and the outcome variables. Pre- and post-testing covered an analysis of each participant’s body composition and six motor performance tests. The 20m sprint was performed at the beginning. Afterward, the tasks lateral jumping from side-to-side, push-ups, and sit-ups were completed in circuit mode. Finally, the 30–15 Intermittent Fitness Test (30–15_IFT_) was performed. Only the 30–15_IFT_ was performed with the entire group at the same time, while all other tests were conducted individually.

The pre-test (baseline testing) was performed 120h before the first training session, the post-intervention testing 72h after the final training session. Both tests were carried out at the same time of day. The testing was monitored and coordinated by the scientific project manager and the four test assistants. The latter have received standardized training instructions, and each supervised a fixed task. The measurement of the body composition was conducted indoors, while all tests for motor performance were performed outdoors on the sports field belonging to the detention house. It was dry during both tests, and the outside temperature was approximately 8°C (46.4°F). Prior to testing motor performance, a warm-up was conducted with the participants. Two days before the pre-test the inmates were informed and made familiar with the testing procedure.

### The Intervention

The training program included strength and endurance elements by combining static and dynamic strength exercises and running related movements. Equivalent to the training characteristics of HIIT, the present program involved modules targeting an all-out intensity in both muscular endurance and endurance exercises, meaning the maximum number of repetitions and the maximum covered distance within each given interval, respectively. For more information about the training program see Table 1.

**Table 1:** Training program.

Week	Session	Training program (work/rest)	
1	1	Five sets of 30/30s Burpees Skippings Superman Squats Leg levers Push-ups	
1	2	Three sets of varying intervals Skippings (30/30s) Burpees/Leg levers (30/30s) Lunges/Isometric squat (60/60s) Rowing (45/60s) Plank (20/10s) x 8	
1	3	Six sets of 30/30s Burpees Squats Sit-ups Push-ups Running	
2	1	Three sets of 45/30s Lunges Isometric squat Skippings Rowing Push-ups Sit-ups Running	
2	2	Four sets of 30/30s Burpees Jumping jacks Leg levers Lunges Superman Squats Push-ups Running	
2	3	Part I: seven sets of 20/10s Burpees Lunges Push-up-Plank-Flow Sit-ups	Part II: 10 sets of 30s/60s Running
3	1	Part I: Three sets of 60/30s Rowing Burpees Squats Skippings Iron mikes Running	Part II: “Bring Sally up”-Challenge Push-ups (03:25min.)
3	2	Part I: four sets of 30/15s Burpees Superman Rowing Mountain climbers Push-ups Isometric squat Skippings	Part II: 12 sets of 30s/15s Running
3	3	Five sets of 30/15s Jumping jacks Iron mikes Push-ups Sit-ups Burpees Leg levers Hip thrusts Running	
4	1	Five sets of 40/20s Skippings Burpees Superman Push-ups Squats Running	
4	2	Part I: six sets of 15/15s Burpees Iron mikes Mountain climbers Push-ups Skippings Squats Jumping jacks Lateral side-to-side jumps	Part II: 12 sets of 15s/15s Running
4	3	Part I: five sets of 30/15s Burpees Jumping jacks Squats Mountain climbers Sit-ups Lateral side-to-side jumps Running	Part II: “Sally-Tabata”-Challenge Push-ups (04:02min.)
5	1	Five sets of 40/20s Skippings Jumping jacks Lunges Lateral side-to-side jumps Squats Runnings	
5	2	Part I: three sets of 20/10s Squats Lunges Skippings Mountain climbers Push-ups Plank Leg levers Sit-ups	Part II: 18 sets of 40s/20s Running
5	3	Part I: one set of 30/30s Burpees Lateral side-to-side jumps Mountain climbers Sit-ups Push-ups	Part II: 4 sets of 4min./3min. Running
6	1	Part I: six sets of 20/10s Squats Burpees Skippings Mountain climbers Push-ups Lateral side-to-side jumps	Part II: 12 sets of 30s/30s Running
6	2	Seven sets of 15/15s Skippings Squats Iron mikes Mountain climbers Push-ups Sit-ups Lateral side-to-side jumps Running	Part II: “The Champ is here”-Challenge Burpees/Skippings (02:36min.)
6	3	Part I: three sets of 30/10s Lateral side-to-side jumps Squats Lunges Burpees Sit-ups Push-ups	Part II: 12 sets of 15s/30s Running

The strength exercises included burpees, squats, lunges, iron mikes, and mountain climbers. These whole-body exercises were chosen because they require no equipment and target several large muscle groups. To perform these dynamic exercises with a high repetition rate, strength, muscular endurance, and endurance were required. In further distinction to Sperlich et al. (2017), all running parts were also performed in interval form.

Every session was performed outdoors on the penitentiary’s sports field and had duration of approximately 30min excluding the warm-up phase. The training was led by two experienced coaches. While one coach demonstrated the exercise, the other coach gave corrections and assistance. The training sessions were not supervised by an employee of the prison. The warm-up program took approximately 10min and was designed to prepare the test persons adequately for the actual training and to minimize the risk of injuries. In each session, the warm-up program involved running based exercises stimulating the overall musculature.

Since the participants had work or school obligations during the day, every training session took place in the evenings at 7pm. During the training, music desired by the participants was played continuously. The selected music, mostly hip hop, had a motivating influence on the participants. Some sessions ended by the so called “Bring Sally up” challenge (Dransmann, 2019), the “Sally (Tabata)”-challenge, or the “The Champ is here”-challenge. All three challenges were music based and required the performance of specific exercises in a given rhythm. The melody and lyrics of the songs provide the load structure (lift and lower or work and rest) for the exercises. The entertaining character loosened up the training and allowed inmates to have small competitions in the training. The competitions satisfied the obvious need of some participants to demonstrate physical superiority or power (Jewkes, 2005).

The participants’ heart rate was monitored throughout every training session continuously with a chest strap and a heart rate monitor (Polar F1, Polar Electro, Oy, Finland). The measured heart rates were used to monitor and evaluate the level of physical exertion during and after the training. The average and maximum heart rate of each participant was documented after each session in front of the entire training group. This public measurement was chosen to create motivation to outperform themselves and others in terms of exertion. In addition, the participant with the highest average heart rate (as a percentage of maximum heart rate) was allowed to select the music for the next session. The average heart rate was chosen so that the intensity should be as high as possible during the entire training. The music was chosen by the participants themselves: sport programs in prisons are one of the few niches in a totalitarian institution in which inmates can temporarily experience themselves as self-determined persons (Müller and Mutz, 2019).

### Anthropometric Data and Body Composition

Various anthropometric parameters were assessed in the pre- and post-test. Specifically, body height was measured using a stadiometer (Seca 213, Seca, Germany) with the participants standing barefoot. All data capturing body composition (i.e., body mass, BMI, fat mass, and fat-free mass) were measured with an eight-electrode bio-impedance scale (Inbody 270, Inbody, Germany). Since dehydration might influence the bio-impedance analysis, participants were asked to consume 500ml of water 1h before measurement. In order to have dietary habits regulated and equalized on testing days, the test persons were instructed to eat their last main meal 2h before measurement at the latest.

### Motor Performance

#### 20m Sprint

The 20m sprint was performed to test the maximum running speed of participants. The objectivity of the 20m sprint was improved by using optical light barriers (Lichtschranke PR1aW, ALGE-Timing GmbH, Germany) for timing. During the set-up, the start line was moved back 1m to avoid premature triggering of the light barrier (Bös and Schlenker, 2016). The sprint was performed twice by each participant and the best time from both trials (in seconds to the exact tenth of a second) was taken as the measured value.

#### Standing Long Jump

The standing long jump was performed to give information about the level of explosive strength and the jumping power of participants, respectively. The jump was performed twice by each participant and the best value from both trials (in centimeters) was taken as the measured value.

#### Lateral Jumping From Side-to-Side

Lateral side-to-side jumps were used as an indicator of coordinative abilities under time pressure. The participants were asked to jump from one half of a 50cm×100cm rectangle without overstepping the lines or having contact with the centerline to the other half as frequently as possible within a time limit of 15s. The lateral side-to-side jumps were performed twice by each participant, and the mean value from both trials (as the number of repetitions) was taken as the measured value.

#### Testing Muscular Endurance

To test muscular endurance, participants were asked to perform as many push-ups and sit-ups as possible within the time limit of 40s. The correct execution was taken from the German motor test 6–18 (Deutscher Motorik-Test; DMT; Bös and Schlenker, 2016). Each push-up begins in the prone position, while hands touch each other on the buttocks. Hands must then be placed next to the shoulder. When the arms are stretched and the body is released from the floor, one hand is released from the floor and touches the other hand. Then the arms are bent until the body is back in the starting position. During a sit-up, the participant must raise the upper body from a lying position and touch both knees with both elbows. When putting the upper body down, both shoulder blades must touch the mat. Both tasks were performed once by each participant and the number of correctly performed repetitions was taken as the measured value.

#### The 30–15 Intermittent Fitness Test

The 30–15 Intermittent Fitness Test is a level test used to measure general aerobic and anaerobic endurance performance. It consists of running intervals that comprise 30s of activity and are separated by 15s of passive regeneration phases. The given velocity in the first interval is 8km/h. In every new interval, the velocity increases by 0.5km/h. Within 30s of running, participants are supposed to move from one line to a second line, with the tow lines being 40m apart from each other. The test ends when the participant is totally exhausted and volitionally stops or if the participant is unable to reach the next zone at the beep on three successive occasions. The running velocity during the last completed stage was taken as the maximum running speed (V_IFT_). Consequently, the 30–15_IFT_ can be considered a maximum load test method to gain information about aerobic and anaerobic performance of participants (Buchheit, 2008).

The review of Grgic et al. (2020) shows that the 30–15_IFT_ has an excellent test–retest reliability for maximum running speed and maximum heart rate. Therefore, participants carried a chest strap and a heart rate monitor (Polar F1, Polar Electro, Oy, Finland) during the test. At the end of the test, the maximum heart rate was documented by the tests assistants.

The maximum oxygen uptake (VO_2max_) can be derived from the V_IFT_. Estimated VO_2maxIFT_ was calculated from V_IFT_ and the athlete’s gender (G), age (A), and body mass (BM) as follows (Buchheit et al., 2011):


$$\begin{array}{c} {\mathrm{VO}}_{\mathrm{2maxIFT}}\;\left(\mathrm{ml}/\min/\mathrm{kg}\right)=28.3-2.15G-0.74\mathrm{1A}-\\ 0.0357\mathrm{BM}+0.05\mathrm{8A}\;x\;V_{\mathrm{IFT}}+1.03V_{\mathrm{IFT}}. \end{array}$$

### Statistical Analyses

The empirical analyses were carried out in SPSS 25 and consisted of two main steps. First, descriptive statistics of all variables were obtained, including mean and SD. Second, ANOVAs were estimated to answer the research question. One requirement for conducting ANOVA is the normal distribution of outcome variables. All variables resulting from the anthropometric measurements and the physical performance tests represent the outcomes in the present study and were, therefore, tested for normal distribution using the Shapiro–Wilk test. The results of this test indicate that all variables were normally distributed (*p*>0.05). A repeated-measures ANOVA was performed for each outcome variable (see Figure 1), with measurement point (pre-test or post-test) representing the time factor. An alpha level of 0.05 was used for all statistical tests. In addition to statistical significance, effect sizes were reported using partial eta-square (*η_p_*^2^). According to Cohen (1988), *η*^2^=0.01 represents a small effect, *η*^2^=0.06 a medium effect, and *η*^2^=0.14 a large effect.

## Results

The 11 inmates completed 91.92±7.60% of the 18 training sessions. They achieved an average overall intensity of 77.95±5.58% of their individual maximum heart rate.

### Anthropometric Data and Body Composition

Table 2 summarizes the ANOVA results for the anthropometric data and body composition variables. They show that both body mass and BMI declined significantly from the pre-test to the post-test with large effects. On the other hand, the changes in fat mass and fat-free mass were insignificant.

**Table 2:** Anthropometric parameters (means±SD) before and after the intervention (*n*=11).

Parameter	Pre-test	Post-test	*F*	*p*	*η_p_* ^2^
Body mass (kg)	99.41±24.29	98.27±24.30	11.198	0.007^**^	0.528
Body-mass-index (kg·m^−2^)	31.21±7.38	30.85±7.36	12.066	0.006^**^	0.547
Fat mass (kg)	29.69±16.88	29.83±16.43	0.078	0.786	0.008
Fat-free mass (%)	72.10±9.82	71.59±9.27	1.365	0.270	0.120

** *p*<0.01.

### Motor Performance

Table 3 summarizes the pre- and post-values of the motor performance tests. They show that performance in the 20m sprint did not change significantly between the pre-test and the post-test. In the other five tests, significant improvements with large effects occurred. Specifically, participants were able to perform a significantly higher number of lateral side-to-side jumps, push-ups, and sit-ups. Moreover, they jumped significantly further in the standing long jump and increased their maximum running speed in the 30–15_IFT_. The values for maximum heart rate during 30–15_IFT_ indicate that a comparable workload was reached for both tests. The maximum oxygen uptake derived from the V_IFT_ improved significantly between the pre-test and the post-test.

**Table 3:** Motor performances (means±SD) before and after the intervention (*n*=11).

Parameter	Pre-test	Post-test	*F*	*p*	*η_p_* ^2^
20m Sprint (s)	3.34±0.39	3.35±0.34	0.120	0.736	0.012
Lateral side-to-side jumps (n)	41.82±9.50	48.00±4.03	7.057	0.024^*^	0.414
Push-ups (n)	15.09±4.04	19.18±3.97	29.263	<0.001^***^	0.745
Sit-ups (n)	19.82±8.15	26.00±6.18	28.473	<0.001^***^	0.740
Standing long jump (cm)	168.36±35.00	181.36±36.91	19.284	0.001^**^	0.659
V_IFT_ (km/h^−1^)	13.86±2.94	16.09±2.77	30.013	<0.001^***^	0.750
VO_2maxIFT_ (ml/min/kg)	38.58±8.73	44.11±8.10	26.718	<0.001^***^	0.728
HF_maxIFT_ (bpm)	185.73±13.08	186.00±13.16	0.050	0.828	0.005

*** *p*<0.001;

** *p*<0.01;

* *p*<0.05.

## Discussion

The key findings concerning the effects of the 6-week functional HIIT were as follows. Body mass and BMI significantly decreased. Fat mass, fat-free mass, and sprint times did not change significantly. A significantly longer distance was covered in the standing long jump. The number of repetitions in push-ups, sit-ups, and lateral side-to-side jumps increased significantly. Also, the maximum running speed in the 30–15_IFT_ and the maximum oxygen uptake increased significantly.

On average, the body mass decreased by 1.1% and the BMI by 1.2%. In both cases, this decrease corresponds to about half the improvement of Sperlich et al. (2017), where the 11 overweight women trained 3weeks longer with the same frequency (three sessions/week) and a similar session duration (30min). Because not all participants in the present study were overweight and the inmates continued to be exposed to everyday prison life including unhealthy behaviors like physical inactivity, drugs, alcohol, smoking, and violence (Fazel and Baillargeon, 2011), this finding represents a good result. In the context of a prison, maintaining body mass and BMI can already be considered a beneficial outcome. For example, existing research has indicated that maintaining the status quo can be quite challenging. The control group in Battaglia et al. (2013) had a BMI increase of 1.4% after 9months (*n*=18), the control group of Pérez-Moreno et al. (2007) witnessed an increase in body mass of 0.3% after 4months (*n*=10).

The decrease in body weight and BMI cannot be explained through the development of the absolute fat mass and the relative fat-free mass. These changes may have occurred to a significant decrease of the body water from 51.04 (±6.52) to 50.09 (±6.46) L, which represents a large effect (*F*=26.959, *p*<0.001, *η*^2^=0.729).

The sprint times have become 0.3% slower on average. This lack of improvement was likely given that the focus of the training was on muscular and aerobic endurance. To improve speed, different components, such as technique, coordination, and maximum muscular strength should have been featured more prominently in the program. In Wong et al. (2010), the 30m sprint times of professional soccer players improved significantly after 8weeks of running HIIT and muscular strength training (both twice a week). The improvements – opposite to our study – can be explained by the shorter and faster running intervals (16 sprints of 15s at 120% of maximum aerobic speed), and especially the high loads and low number of repetitions in strength training (four sets of six repetitions maximum).

By far the greatest improvements can be seen for both muscular endurance exercises. The number of push-ups increased by 27.1% and the number of sit-ups by 31.2%, with both improvements being statistically significant. Such an improvement was expected for two reasons: both exercises were performed in almost every session and the focus of the training program was on muscular endurance. The improvements were similar to studies in which training was performed more frequently or over a longer time period. For instance, in Sperlich et al. (2018), the group that trained twice daily for 4weeks improved the number of sit-ups in 1min by 29.6%. In Battaglia et al. (2013), inmates in both training groups enhanced their push-up performance after 9months with two sessions per week: the CRT-group by 24%, the HIST-group by 28.1%.

The improvements in muscular endurance cannot be explained by changes in body composition. The gains in muscle protein mass may take several months (Zinner et al., 2017). Consequently, the improvement in muscular endurance likely results from neural adaptation and improvements in intramuscular coordination.

The number of lateral side-to-side jumps increased by 14.8%, the distance in long jump by 7.7%. Even though coordinative abilities under time pressure and high-speed strength were not explicitly trained, the improvements can be explained with the same type of activity (Bös and Schlenker, 2016). The present program included many jumping movements. Side jumps (without spatial restriction) were trained in seven sessions, with other exercises like burpees and jumping jacks also carrying jumping elements in them. Moreover, if possible, elements such as squats and lunges were instructed to be jumped or performed explosively.

The maximum running speed increased by 16.1%. Converting this value into VO_2max_, the average improvement was equivalent to 14.3%. This change was higher than improvements in comparable research: for example, Sperlich et al. (2017) reported only a 10.1% improvement in maximum oxygen uptake, although providing an additional 3weeks of training. The main reason for this difference might be the increased proportion of running within the present training program: In 16 of 18 sessions, inmates had to run. The improvement in VO_2max_ can be explained by better central adaptation, including augmented plasma and blood volumes (Graham et al., 2016) with elevated stroke volume (Goodman et al., 2005).

An intensity of just under 80 percent of the maximum heart rate can be considered relatively high for a mix of strength and endurance training. For example, the corresponding value was 72.6% in Sperlich et al. (2018). Hence, the level of purely endurance based HIITs (90% of maximum heart rate) was not reached in both our study and in Sperlich et al. (2018). These values illustrate a recurring finding: whereas a HIFT protocol resulted in heart rates of around 90% of the age-predicted maximum throughout the whole workout, the average heart rates for a multimodal/functional HIIT protocol were around 76%, with only the peaks for each interval approaching 90% of the maximum heart rate (Butcher et al., 2015). The analysis of the heart rate data suggests that the average heart rates were lowest in sessions 1.2 and 2.1. These were the sessions including a static exercise (isometric squat) and a lying exercise (rowing). In the only session, where these two exercises were included again (3.2), the increased running portion compensated the lower heart rates resulting from static and lying exercises.

The high training participation of about 92% shows how well the training was received in this setting. The adherence was much higher than in comparative prison studies. It was also higher than participation rates in other functional HIIT programs such as 89% in Sperlich et al. (2017) and 88.2% in Sperlich et al. (2018). This high degree of adherence may be attributed to the intervention being relatively short (6weeks) and representing a welcome distraction from inmates’ daily routine. It was also important that the training sessions did not take up too much time, i.e., only about 40min. The inmates in this prison, which is similar to other open prisons in Germany, have a maximum of 5h of free time per day. This period begins with the end of work at around 5 pm and ends with the closing of the cells at 10 pm. Hence, the attractive time-use ratio of HIIT aligns well with the conditions of an open German prison. The only material that was used was gymnastic mats, a music system, and sports watches. Collectively, the present training program only requires few resources in terms of time and equipment and can, therefore, be carried out in any other detention center.

## Limitations and Future Research

The main limitations originate from the setting, i.e., the prison context. The most difficult circumstances were the small sample size and the lack of a control group. Both restrictions resulted from the small number of inmates in this institution. Twenty of the total 40 inmates agreed to participate. This participation rate corresponds to 50%. Of the remaining 20 inmates, no volunteers could be found who agreed to participate in the pre- and post-tests. They could have been obliged by the management of the prison, but due to the lack of motivation, the data for motor performance requiring maximum effort would not have been reliable. The present study shares the limitations of other fitness interventions in prison also relying on a small sample size. The 13 participants in the present study exceeded the two participants of Amtmann and Kukay (2016) by a margin.

Although we instructed our participants not to alter their eating habits, we cannot exclude the possibility that some inmates nevertheless altered their nutritional intake. Because participation in the training already involved a significant restriction of free time, we did not want to overburden the participants with keeping a food diary. However, after consultation with the kitchen management, the menu did not differ regarding protein or carbohydrate intake from the weeks before the pre-test.

Additionally, the non-exercise activity level plays a role in energy consumption. Since the prisoners were only allowed to leave the prison for work, such activity only occurred in the context of work. Because all participants were employed in a craft profession (mainly gardening and kitchen), the non-exercise physical activity was comparable for all inmates.

Drug use can also impact body composition (Hartgens et al., 1996) and motor performance (Singh et al., 1984). Accordingly, not taking drugs was a prerequisite for inclusion in the study. Notably, the use of drugs is generally forbidden for prisoners. In the event of a positive drug test – which were carried out frequently and without previous notice – the inmate would be transferred to a locked facility. Consequently, the inmates would not admit to taking drugs and have an incentive to not take any.

Testing of motor skills was limited to field tests. Even though only tests with a high reliability and validity were selected (Buchheit, 2008; Bös and Schlenker, 2016), laboratory tests would have a higher accuracy. The high improvements suggest that learning effects through test repetitions occurred. Likewise, Bös and Schlenker (2016) showed that the test results in the DMT improved significantly with an average by 6.3% in the retest (without training).

Another limitation is the lack of further cardiovascular data for the training or the 30–15_IFT_, because the Polar FT1 just exported the average and peak heart rate. In future studies, recording heart rate variability could provide additional information about physical stress or psychological strain, especially in everyday prison life. Another avenue for future research is the conduction of a purely running based HIIT or a HIFT. To date, such interventions have not yet been conducted and evaluated in a prison context. Moreover, female inmates should be considered in future sport interventions, as they have received little or no attention to date.

## Data Availability Statement

The raw data supporting the conclusions of this article will be made available by the authors, without undue reservation.

## Ethics Statement

The studies involving human participants were reviewed and approved by Ethics Committee of Bielefeld University. The patients/participants provided their written informed consent to participate in this study.

## Author Contributions

MD, MK, BG, and PW contributed to the conception and design of the study and wrote sections of the manuscript. MD organized the database and performed the statistical analysis. MD and MK planned and led the intervention as well as wrote the first draft of the manuscript. PW copyedited the draft for content and language, and organized the revision process. All authors contributed to manuscript revision, read, and approved the submitted version.

## Funding

We acknowledge the financial support of the German Research Foundation (DFG) and the Open Access Publication Fund of Bielefeld University for the article processing charge.

## Conflict of Interest

The authors declare that the research was conducted in the absence of any commercial or financial relationships that could be construed as a potential conflict of interest.

## Publisher’s Note

All claims expressed in this article are solely those of the authors and do not necessarily represent those of their affiliated organizations, or those of the publisher, the editors and the reviewers. Any product that may be evaluated in this article, or claim that may be made by its manufacturer, is not guaranteed or endorsed by the publisher.
